# A Fast-Response AIE-Active Ratiometric Fluorescent Probe for the Detection of Carboxylesterase

**DOI:** 10.3390/bios12070484

**Published:** 2022-07-03

**Authors:** Mengting Xia, Chunbin Li, Lingxiu Liu, Yumao He, Yongdong Li, Guoyu Jiang, Jianguo Wang

**Affiliations:** 1 Key Laboratory of Organo-Pharmaceutical Chemistry, Gannan Normal University, Ganzhou 341000, China; 1190907030@gnnu.edu.cn (M.X.); ydli2005@gmail.com (Y.L.); 2 College of Chemistry and Chemical Engineering, Inner Mongolia University, Hohhot 010021, China; 22007018@mail.imu.edu.cn (C.L.); 31907001@mail.imu.edu.cn (L.L.); 32007012@mail.imu.edu.cn (Y.H.); jiangguoyu@mail.ipc.ac.cn (G.J.)

**Keywords:** carboxylesterase, hepatocellular carcinoma, aggregation-induced emission, fluorescent probe, ratiometric

## Abstract

Hepatocellular carcinoma (HCC) is associated with a high mortality rate worldwide. The therapeutic outcomes can be significantly improved if diagnosis and treatment are initiated earlier in the disease process. Recently, the carboxylesterase (CaE) activity/level in human plasma was reported to be a novel serological biomarker candidate for HCC. In this article, we fabricated a new fluorescent probe with AIE characteristics for the rapid detection of CaE with a more reliable ratiometric response mode. The TCFISE probe showed high sensitivity (LOD: 93.0 μU/mL) and selectivity toward CaE. Furthermore, the good pH stability, superior resistance against photobleaching, and low cytotoxicity highlight the high potential of the TCFISE probe for application in the monitoring of CaE activity in complex biological samples and in live cells, tissues, and animals.

## 1. Introduction

Hepatocellular carcinoma (HCC), the major form of liver cancers, is associated with a high mortality rate worldwide, with over 662,000 deaths reported annually [1,2]. The therapeutic outcomes can be significantly improved if diagnosis and treatment are initiated earlier in the disease process. Currently, the common screening methods for HCC mainly rely on measurement of the blood levels of α-fetoprotein (AFP), imaging (including ultrasound, CT, and MRI), and biopsy [3,4,5,6,7,8,9,10,11,12]. However, the evaluated levels of AFP are not always reliable, with a sensitivity of 41–65% and specificity of 80–94%, resulting in a high rate of misdiagnosis and missed diagnosis. Ultrasound and CT imaging suffers from low sensitivity, while MRI incurs a high cost [13,14,15,16,17]. Biopsy remains the most reliable method, and it is required to confirm the diagnosis of HCC. However, it is time-consuming and may take several days for patients to receive the final results.

Fortunately, small-molecular fluorescent probes, with the merits of high sensitivity and specificity, facile operation, and noninvasiveness, are emerging as a powerful tool for both fast and reliable diagnosis of various diseases [18,19,20,21,22,23,24,25,26]. Recently, the carboxylesterase (CaE) activity/level in human plasma was reported to be a novel serological biomarker candidate for HCC, as proven in previously reported research results [27,28,29,30,31,32]. Encouraged by this aspect, small-molecular fluorescent probes for CaE have been reported and applied in liver-related imaging and diagnosis [33,34,35,36,37,38,39,40,41,42]. Unfortunately, these probes suffer from the aggregation-caused quenching (ACQ) of fluorescence after their accumulation into cells or aggregation in physiological environments [43,44,45,46]. Aggregation-induced emission luminogens (AIEgens) with the property of a “more aggregate, brighter emission” perfectly circumvent these problems [47,48,49,50,51,52,53,54]. Furthermore, with the unique features of a large Stokes shift, high resistance to photobleaching, and strong brightness, AIEgens are now widely studied and applied in various fields of chemo/biosensing, disease theranostics, and organic light-emitting diodes [55,56,57,58,59,60,61,62]. However, small-molecular fluorescent probes for CaE based on AIEgens are reported in small quantities [32,63,64]. New probes with AIE features are still in high demand to make full use of their advantages.

**Scheme 1:**
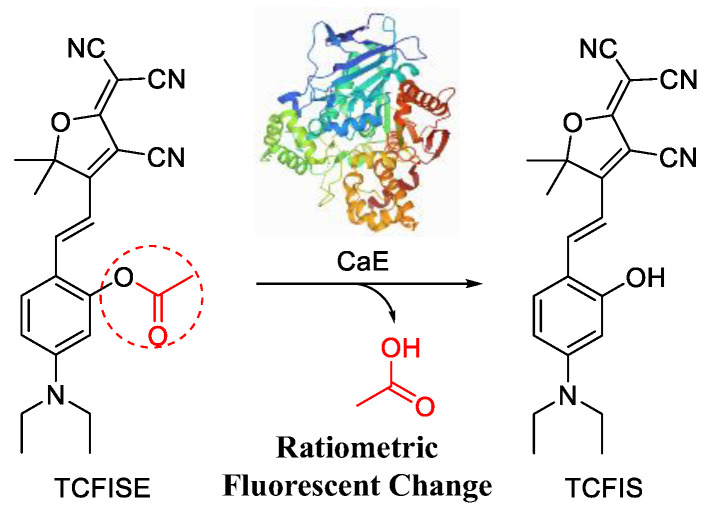
Schematic illustration of the ratiometric response of AIE-active TCFISE probe toward CaE.

Here, in this article, a new small-molecular fluorescent probe (TCFISE) for the rapid and reliable ratiometric measurement of CaE was designed and synthesized. In comparison to probes with an “on/off” or “off/on” photoluminescence (PL) intensity response relying on a single emission wavelength, ratiometric AIE-active probes can provide PL intensity ratio changes on the basis of two separate emission wavelengths, which are favorable for avoiding interference due to probe concentration, temperature, pH, and polarity, thus potentially providing more reliable test results. As displayed in Scheme 1 and Scheme S1, the TCFISE probe can be easily obtained through a two-step reaction process. First, a Knoevenagel condensation reaction between 2-(3-cyano-4,5,5-trimethylfuran-2(5H)-ylidene)malononitrile (TCF) and 4-(diethylamino)salicylaldehyde results in TCFIS with strong donor–acceptor (D–A) interactions, which is favorable for extending the emission of the probe to longer wavelengths [65,66,67,68,69]. Then, a CaE responsive acetyl group is attached to TCFIS to obtain the TCFISE probe. Spectral data proved that both TCFIS and the TCFISE probe were AIE-active. TCFISE exhibited bright-red emission at 651 nm and showed a rapid response toward CaE with a ratiometric mode. The PL intensity ratio at 629 nm vs. 651 nm demonstrated a good linear relationship with the concentration of CaE, resulting in a detection limit of 93.0 μU/mL, indicating the high sensitivity of the TCFISE probe. Furthermore, the TCFISE probe also displayed high selectivity toward CaE over common metal ions, amino acids, anions, reactive oxygen and nitrogen species (ROS and RNS), and enzymes in a biological environment. These features, along with its high stability in different buffer solutions with varied pH values, high resistance against photobleaching, and negligible cytotoxicity, make TCFISE a powerful fluorescent probe for CaE with potential application in the early diagnosis and therapy of HCC.

## 2. Materials and Methods

### 2.1. Materials and Instrumentation

Chemicals were purchased from Admas, Innochem, leyan, Sigma-Aldrich and used without further purification. Carboxylesterase (CaE) from porcine liver was purchased from Sigma-Aldrich. Double-distilled water was supplied by the Milli-Q Plus System (Millipore Corporation, Bedford, MA, USA).

^1^H-NMR and ^13^C-NMR spectra were measured on a Bruker ARX 500 MHz spectrometer. UV/Vis absorption spectra were recorded on a UV-2600i UV/Vis spectrophotometer. Fluorescence emission spectra were recorded on a FL-4700 fluorescence spectrophotometer. High-resolution mass spectra were collected on a Waters G2-Xs QTOF high-resolution mass spectrometer.

### 2.2. Synthesis and Characterization of TCFISE Probe

The synthetic route is shown in Scheme S1 in the Supplementary Materials. The structures of all compounds were confirmed by NMR and HRMS (Figures S1–S6 in the Supplementary Materials).

Synthesis of TCFIS. A mixture of 4-(diethylamino)salicylaldehyde (compound **1**, 200.0 mg, 1.04 mmol), 2-(3-cyano-4,5,5-trimethylfuran-2(5H)-ylidene)malononitrile (compound **2**, 247.4 mg, 1.24 mmol), and ammonium acetate (119.75 mg, 1.55 mmol) in ethanol (15 mL) was refluxed under a nitrogen atmosphere overnight. Then, the mixture was cooled to room temperature and purified by silica gel chromatography with petroleum ethyl/dichloromethane (1:5, *v*/*v*) to give TCFIS as a purple solid (280.0 mg, 72.3%). ^1^H-NMR (500 MHz, DMSO) *δ* 10.84 (br, 1H), 8.21 (br, 1H), 7.69 (d, *J* = 9.2 Hz, 1H), 6.93 (d, *J* = 15.3 Hz, 1H), 6.44 (dd, *J* = 9.3, 2.4 Hz, 1H), 6.14 (d, *J* = 2.4 Hz, 1H), 3.44 (q, *J* = 7.1 Hz, 4H), 1.68 (s, 6H), 1.14 (t, *J* = 7.0 Hz, 6H). ^13^C-NMR (126 MHz, DMSO) *δ* 177.96, 175.71, 162.61, 154.51, 114.54, 113.66, 113.29, 112.41, 107.24, 97.65, 96.86, 49.21, 45.02, 26.40, 13.12. HRMS (MALDI-TOF): *m*/*z*: [M + H]^+^ calculated for C_22_H_23_N_4_O_2_^+^: 375.1821; found: 375.1824.

Synthesis of TCFISE. A mixture of acetylchloride (62.5 mg, 0.8 mmol), triethylamine (6 μL, 0.08 mmol), and TCFIS (200 mg, 0.53 mmol) in 10 mL of dry CH_2_Cl_2_ was stirred at −20 °C for 4 h. The reaction mixture was purified by silica gel chromatography using petroleum ether/ethyl (3:1, *v*/*v*), and TCFISE was obtained as a blue to purple solid (143.9 mg, 62.1%). ^1^H-NMR (500 MHz, DMSO) *δ* 8.08 (d, *J* = 15.9 Hz, 1H), 7.65 (d, *J* = 9.2 Hz, 1H), 6.62 (dd, *J* = 9.1, 2.6 Hz, 1H), 6.57 (d, *J* = 15.9 Hz, 1H), 6.39 (d, *J* = 2.6 Hz, 1H), 3.48 (q, *J* = 7.2 Hz, 4H), 2.44 (s, 3H), 1.70 (s, 6H), 1.26 (t, *J* = 7.1 Hz, 6H). ^13^C-NMR (126 MHz, CDCl_3_) *δ* 176.65, 174.24, 169.11, 153.61, 152.94, 142.21, 129.81, 114.04, 112.80, 112.21, 112.02, 110.47, 108.34, 105.22, 97.01, 45.20, 26.31, 21.18, 12.61. HRMS (MALDI-TOF): *m*/*z*: [M + H]^+^ calculated for C_24_H_25_N_4_O_3_^+^: 417.1927; found: 417.1931.

### 2.3. General Procedures for the Detection of CaE

Unless otherwise noted, all the spectral measurements were performed in 1 mM phosphate buffer (pH = 7.4) according to the following procedure: CaE was prepared in aqueous solution. The stock solution (1.0 mM) of the TCFISE probe was first prepared in DMF. Then, 10 μL of TCFISE stock solution was added to 1.4 mL of PBS and 0.6 mL of DMF. CaE was added, and the mixture was incubated for different times at 37 °C. Then, the reaction solutions were transferred to a quartz cell with 1 cm optical length for measurements.

### 2.4. Limit of Detection (LOD) for TCFISE toward Addition of CaE

According to the linear, the LOD was estimated as follows:

LOD = 3σ/B,(1)

where σ is the standard deviation obtained from the individual fluorescent intensity ratio (*I*_629_/*I*_651_) of TCFISE (5 μM) without any CaE, and B is the slope obtained after linear fitting of the titration curves within certain ranges.

### 2.5. Cell Culture and Cell Viability

The HeLa cells were cultured in DMEM (containing 10% heat-inactivated FBS, 100 mg∙mL^−1^ penicillin, and 100 mg∙mL^−1^ streptomycin) at 37 °C in a humidified incubator with 5% CO_2_. Before the experiments, the cells were precultured until confluence was reached.

Cell viability was determined using the MTT assay, which is based on the reduction of 3-(4,5-dimethythiazol-2-yl)-2,5-diphenyl tetrazolium bromide (MTT, yellow in color) into formazan (blue color) by mitochondrial succinate dehydrogenase. First, 100 μL of cell suspension (5000 cells/well) was dispensed in a 96-well plate. The plate was preincubated for 24 h at 37 °C in a humidified incubator with 5% CO_2_. Then, 10 μL of TCFISE at various concentrations was added into the culture media in the plate. After incubating the plate for 20 h in the incubator, the cell medium was exchanged with fresh medium (100 μL) containing 20 μg/mL MTT. The medium was removed after an incubation period of 4 h, followed by the addition of 100 μL of DMSO to dissolve the formazan crystals. Absorbance was taken at 595 nm by an ELISA Plate Reader (Biotek Synergy HT). Untreated cells were taken as the control. All experiments were performed in triplicate. Cell viability was determined using the following formula:

(2) $$\mathrm{Cell}\;\mathrm{viability}\;(\%)=\frac{\mathrm{Absorbance}\;\mathrm{of}\;\mathrm{treated}\;\mathrm{cells}}{\mathrm{Absorbance}\;\mathrm{of}\;\mathrm{untreated}\;\mathrm{cells}}$$

### 2.6. Cell Imaging

The HepG2 cells were incubated with 5 μM TCFISE and 1 μM Hoechst in DMEM containing 5% DMSO at 37 °C for 30 min, and then washed three times with PBS (pH 7.4). Further experiments were carried out by pretreating cells with BNPP (0, 20, 50, and 100 μM). Briefly, the cells were firstly washed three times with DMEM (without FBS), and then incubated with BNPP for 1.5 h. After that, the cells were washed three times with PBS, and then incubated with TCFISE and Hoechst. The cells were viewed using a Nikon eclipse Ti2-A inverted microscope. For TCFISE, a 500–580 nm laser light was used as the light source, and the emission at >594 nm was collected. For Hoechst, a 352–402 nm laser light was used as the excitation light, and the emission was collected in the range of 417–477 nm.

## 3. Results and Discussion

### 3.1. AIE Characteristics of the TCFISE Probe and TCFIS

**Figure 1:**
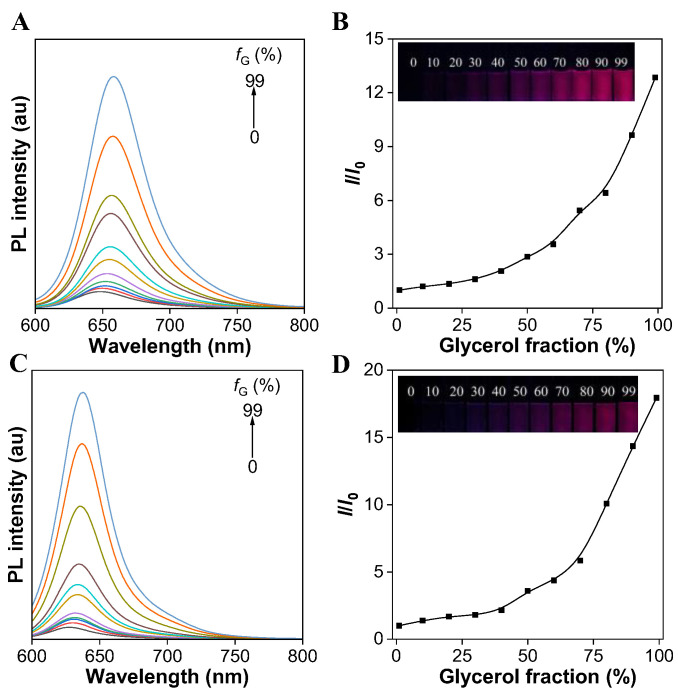
(**A**) PL spectra of TCFISE (5 μM) in methanol/glycerol mixed solution with different glycerol fractions (*f*_G_). (**B**) Plot of relative PL intensity (651 nm) of TCFISE (5 μM) versus glycerol fraction. The inset shows the fluorescent images of TCFISE in solutions with different *f*_G_ taken under 365 nm UV light. (**C**) PL spectra of TCFIS (5 μM) in methanol/glycerol mixed solution with different *f*_G_. (**D**) Plot of relative PL intensity (629 nm) of TCFISE (5 μM) versus glycerol fraction. The inset shows the fluorescent images of TCFIS in solutions with different *f*_G_ taken under 365 nm UV light. λ_ex_ = 575 nm.

To verify the AIE feature, the PL spectra of both the TCFISE probe and TCFIS were recorded in methanol solution containing different volume fractions (*f*_G_) of glycerol. As shown in Figure 1A,B, the TCFISE probe exhibited dim emission in pure methanol solution, while the PL intensity of the TCFISE probe was enhanced gradually with the addition of glycerol and reached its maximum when the *f*_G_ reached 99%. TCFIS displayed similar behaviors in the methanol/glycerol mixed solution (Figure 1C,D). These data indicated that both the TCFISE probe and TCFIS exhibit AIE characteristics, according to previous reports [44,45,46]. Since glycerol is a viscous solvent, which is much more viscous than methanol, the addition of glycerol would gradually increase the viscosity of the mixed solution. According to the restriction of intramolecular motion (RIM) mechanism of typical AIEgens, in methanol with low viscosity, the exciton energy could be efficaciously dissipated by the active intramolecular motion of TCFISE or TCFIS, thus leading to weak emission. On the other hand, in highly viscous solvents, these intramolecular motions could be suppressed, thus opening up the emission channel, which resulted in bright emission in the mixed solution with high *f*_G_. The AIE feature of both TCFIS and the TCFISE probe was also verified in a DMSO/toluene mixed solution with different toluene fractions (*f*_T_). The results in Figure S7 display the gradually enhanced emission intensity with the increase in *f*_T_. The PL intensity reached a plateau at *f*_T_ of 90% for TCFIS and 80% for TCFISE. Their emission intensity probably decreased due to the change in aggregate state, as also reported for other AIEgens. The AIE feature of both TCFIS and the TCFISE probe is favorable for their application in biological fields, since AIEgens usually show high photostability and high brightness even after entering cells.

### 3.2. Response of TCFISE Probe to CaE

**Figure 2:**
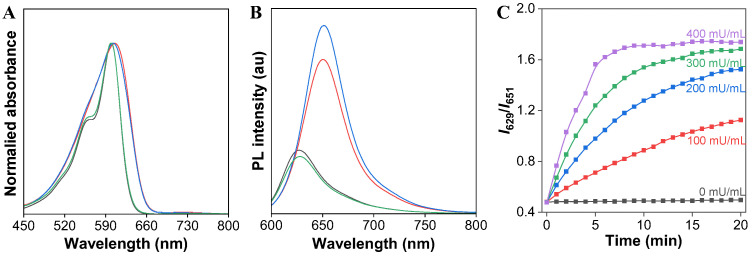
(**A**) Normalized absorption and (**B**) PL intensity spectra of TCFIS (black), TCFISE (blue), TCFISE + CaE (green), and TCFIS + BNPP + CaE (red) in DMF/PBS (3/7, *v*/*v*, pH = 7.4). (**C**) The relative PL intensity ratios (*I*_629_/*I*_651_) of TCFISE in the presence of different amounts of CaE (0–400 mU/mL) as a function of incubation time. Concentrations: TCFISE and TCFIS, 5 μM; CaE, 1 U/mL; BNPP, 1 mM. *λ*_ex_ = 575 nm.

To evaluate our design concept for the ratiometric detection of CaE, we then examined the UV/Vis absorption and PL spectra of the AIE-active fluorescent TCFISE probe before and after incubation with CaE. As depicted in Figure 2, the TCFISE probe exhibited an absorption maximum at 602 nm and a red emission at 651 nm. After incubation with CaE, TCFISE displayed a slightly blue-shifted absorption maximum at 600 nm with a shoulder peak at 563 nm. In comparison with TCFIS, the TCFISE probe showed very similar absorption spectra to those of TCFIS, indicating that the enzymatic reaction product might be TCFIS. To confirm this, the PL spectra of the TCFISE probe incubated with CaE were also compared with those of TCFIS. As displayed in Figure 2B, the PL spectra of TCFISE showed a hypochromatic effect with emission maxima blue-shifted from 651 nm to 629 nm after incubation with CaE, which was in good agreement with those of TCFIS, confirming the enzymatic reaction product. These spectraal data underlined that the TCFISE probe can be utilized for the detection of CaE with a reliable ratiometric mode. Furthermore, in the presence of bis(4-nitrophenyl)-phosphate (BNPP), a well-known inhibitor of CaE, both the absorption and the PL spectra of TCFISE remained unchanged, indicating the inhibition of the CaE activity, which also demonstrated that the spectral changes of TCFISE in the presence of CaE were indeed caused by the enzymatic catalytic hydrolysis of the TCFISE probe to produce TCFIS. This enzymatic hydrolysis reaction was then further confirmed by high-resolution mass spectrometry (HRMS). As shown in Figure S8, after incubation with CaE, the molecular ion peak of TCFISE disappeared, and, instead, a molecular ion peak with *m*/*e* = 375.1819 appeared, which is in accordance with the molecular ion peak of [TCFIS + H]^+^. Then, we tried to follow the reaction time courses of the TCFISE probe with different concentrations of CaE by tracking the PL intensity ratios (*I*_629_/*I*_651_) changes. Remarkably, the ratios of TCFISE exhibited a rapid enhancement in the initial 5 min and leveled off after 20 min, whereas, for the TCFISE probe without CaE, the ratios remained unchanged after 20 min. These data manifested that the probe was highly stable in aqueous solution, and that the response of the probe toward CaE was fast and effective. Accordingly, a time period of 20 min was selected for subsequent experiments.

**Figure 3:**
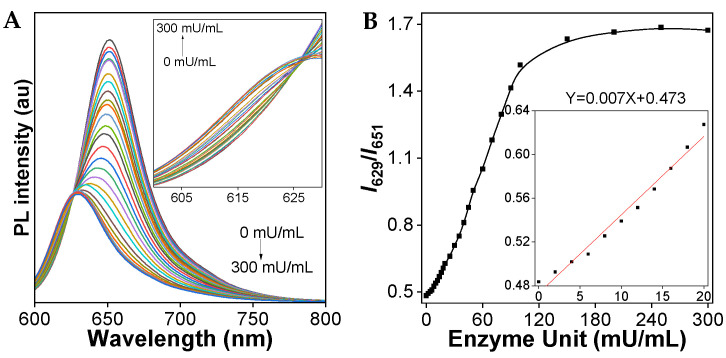
(**A**) PL spectra of TCFISE (5 μM) upon the addition of CaE at 37 °C for 20 min. The inset shows the PL spectra in the range of 600–630 nm. (**B**) Relative PL intensity ratios (*I*_629_/*I*_651_) of TCFISE (5 μM) as a function of CaE concentration. The inset shows the plot and linear fitting of relative PL intensity ratios (*I*_629_/*I*_651_) vs. the concentration of CaE (0–20 mU/mL). *λ*_ex_ = 575 nm.

To examine the sensitivity of the TCFISE probe toward CaE, the PL spectra upon the addition of increasing concentrations of CaE were recorded. As demonstrated in Figure 3, with increasing aliquots of CaE, the PL intensity at 651 nm rapidly decreased, accompanied by a slow enhancement of the PL intensity at 629 nm. The PL intensity ratios of *I*_629_/*I*_651_ displayed a good linear relationship with the concentration of CaE in the range of 0–20 mU/mL, resulting in a limit of detection (LOD) of 93.0 μU/mL, which is sensitive enough to support the clinical diagnosis of HCC, considering that the serum CaE activity/level in patients with HCC is higher than that in healthy human plasma, which is reported to be 0.019 ± 0.001 U/mL [64].

**Figure 4:**
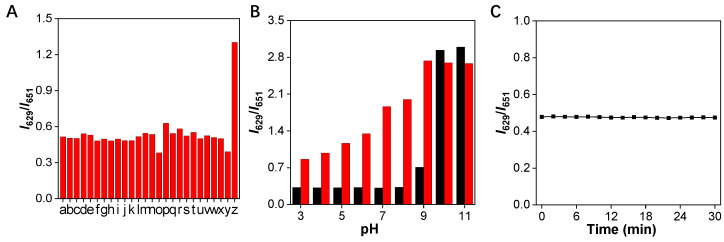
(**A**) Variation of the relative PL intensity ratios (*I*_629_/*I*_651_) of TCFISE (5 μM) after incubation with 200 mU/mL of CaE and other biologically relevant substances. a: AcO^−^ (20 mM); b: Al^3+^ (20 mM); c: Ca^2+^ (20 mM); d: Cu^2+^ (20 mM); e: Fe^3+^ (20 mM); f: K^+^ (20 mM); g: Mg^2+^ (20 mM); h: Na^+^ (20 mM); i: NH_4_^+^ (20 mM); j: NO_2_^−^ (20 mM); k: ClO^−^ (2 mM); l: H_2_O_2_ (2 mM); m: O_2_^−^ (2 mM); n: Vc (2 mM); o: BSA (100 μg/mL); p: Cys (10 mM); q: GSH (10 mM); r: Hcy (10 mM); s: l-glutamine (10 mM); t: Pro (10 mM); u: tyrosinase (200 mU/mL); v: acid phosphatase (200 mU/mL); w: lipopolysaccharide (50 μg/mL); x: glucose (10 mM); y: acetylcholine esterase (200 mU/mL); z: CaE (200 mU/mL). (**B**) Relative PL intensity (*I*_629_/*I*_651_) of TCFISE (black bars) and TCFISE + CaE (red bars) in buffers of different pH. (**C**) Photostability of TCFISE. λ_ex_ = 575 nm.

Specificity is another significant parameter for a good fluorescent probe. Therefore, the specificity of the TCFISE probe toward CaE was further investigated. As shown in Figure 4A, the relative PL intensity ratios (*I*_629_/*I*_651_) remained barely changed in the presence of common cationic and anionic ions, amino acids, redox substances, and enzymes in biological systems. In stark contrast, only the addition of CaE could induce an obvious enhancement of the PL intensity ratio, indicating that the TCFISE probe is highly selective toward CaE, which is favorable for the reliable detection of CaE in serum and urine samples.

Additionally, to further assure the reliable detection of CaE in complex biological samples whose pH values may vary in a wide range, the pH stability of the TCFISE probe in the absence and presence of CaE was assayed. As depicted in Figure 4B, the TCFISE probe exhibited high stability in the absence of CaE in pH values from 3.0–8.0. A higher pH than 9.0 could induce the hydrolysis of the acetyl group in the TCFISE probe. The response of TCFISE toward CaE also showed similar pH stability to the probe itself. The poor responsiveness of TCFISE toward CaE in solutions with pH values above 9.0 could be ascribed, on one hand, to the instability of the probe and, on the other hand, to the diminished activity of CaE in alkaline conditions.

AIEgens always display superior resistance against photobleaching, which is favorable for their application for long-term tracking. Thus, the photostability of the TCFISE probe was also tested. As shown in Figure 4C, the PL intensity ratios displayed negligible variations even over a long time period of 30 min of light irradiation (300–1100 nm, 60 mW/cm^2^, from a CEL-S500 Solar stimulator), indicating its excellent photostability, comparable to other reported AIEgens [51,52,53,54,55,56,57,58].

### 3.3. CaE Measurement in Diluted Serum Samples

To test the possibility of CaE measurement in serum samples using the TCFISE probe, fetal bovine serum (20-fold diluted) was added to the test samples before spiking with different concentrations of CaE. As shown in Figure S9, the PL intensity ratio (*I*_629_/*I*_651_) of the sample with FBS exhibited an enhancement compared to the control sample without FBS, indicating that the FBS contained CaE. Moreover, the PL intensity ratios (*I*_629_/*I*_651_) of TCFISE displayed good linearity with the concentration of the spiked CaE in these diluted serum samples, indicating the potential of CaE measurement in serum samples using our probe, further verifying that our probe could support the clinical diagnosis of HCC.

### 3.4. Cell Cytotoxicity

To verify the application potential of the TCFISE probe for the detection and imaging of CaE in live cells, tissues, or even live animals, the cytotoxicity toward HeLa cells and HepG2 cells was examined using MTT assays. The results can be found in Figures S10 and S11. Cell viability remained unaffected for TCFISE concentrations up to 35 μM, indicating good biocompatibility and the potential cell imaging ability of the probe.

### 3.5. Cell Imaging

To verify the cell imaging capability, the TCFISE probe was incubated with HepG2 cells. As demonstrated in Figures S12 and S13, red emissions were clearly observed inside cells. Commercially available nucleus indicator Hoechst also displayed obvious blue emission inside cells. Moreover, to confirm that the red emission was indeed from the reaction of the TCFISE probe with cellular endogenous CaE, BNPP was used further for cell imaging. As displayed, the red emission inside cells gradually decreased as the concentration of BNPP increased, indicating a dose-dependent effect. These results indicated that red emission inside cells indeed came from the enzymatic reaction of the TCFISE probe and endogenous CaE inside HepG2 cells, proving the cell imaging ability of the TCFISE probe, which may further help for the diagnosis of HCC. We noted that the emission variation of TCFISE after reacting with endogenous CaE was different from that of in DMF/PBS (3/7, *v*/*v*, pH = 7.4) mixed solution. We infer that this may be attributed to the quite different environments between DMF/PBS mixed solution and cellular environments, where a large amount of biomacromolecules such as DNA and proteins may affect the AIE behavior of our TCFISE probe. Inside live HepG2 cells, our probe bound to CaE and underwent an enzymatic reaction. Thus, the TCFIS product could also bind to CaE after the reaction, thus resulting in the enhanced emission observed in Figure S12 inside live HepG2 Cells.

## 4. Conclusions

Collectively, a new small-molecular fluorescent probe (TCFISE) with AIE features was designed for the rapid detection of CaE with a more reliable ratiometric response mode. TCFISE with D–A interactions exhibited bright-red emission in aqueous solution and displayed high sensitivity toward CaE with an LOD value of 93.0 μU/mL. Furthermore, its superior specificity, excellent pH stability, and photostability make TCFISE highly promising for the detection of CaE in complex serum and urine samples. In addition, the good compatibility of TCFISE toward cells provides great potential for its application as an efficient fluorescent imaging agent for the direct observation of CaE activity in live cells, tissues, and animals.

## Data Availability

Not applicable.

## Supplementary Materials

The following supporting information can be downloaded at https://www.mdpi.com/article/10.3390/bios12070484/s1: Scheme S1. Synthetic route to TCFIS and TCFISE; Figure S1. ^1^H-NMR spectrum of TCFIS in DMSO-*d*_6_; Figure S2. ^13^C-NMR spectrum of TCFIS in DMSO-*d*_6_; Figure S3. HRMS spectrum of TCFIS; Figure S4. ^1^H-NMR spectrum of TCFISE in CDCl_3_; Figure S5. ^13^C-NMR spectrum of TCFISE in CDCl_3_; Figure S6. HRMS spectrum of TCFISE; Figure S7.: AIE curve of TCFIS and TCFISE in toluene/DMSO mixed solution with different toluene fractions (*f*_T_); Figure S8. HRMS spectrum of TCFISE after incubation with CaE (1 U/mL) at 37 °C for 20 min; Figure S9. CaE measurement in serum samples using TCFISE probe; Figure S10. Cell viability of TCFISE toward HeLa cells using MTT assays; Figure S11. Cell viability of TCFISE toward HepG2 cells using MTT assays; Figure S12: HepG2 cell imaging of TCFISE in the presence of different concentrations of BNPP; Figure S13: The average intensity of TCFISE channel in the presence of different concentrations of BNPP in Figure S12.
