# Supplementary material for: A Fast-Response AIE-Active Ratiometric Fluorescent Probe for the Detection of Carboxylesterase
Source: Biosensors (Basel). 2022 Jul 3;12(7):484. doi: 10.3390/bios12070484 (PMC9313056; doi:10.3390/bios12070484)
Supplement: Supplementary file 1 [file biosensors-12-00484-s001.zip › biosensors-1804579-supplementary.pdf]

Article

A Fast-Response AIE-Active Ratiometric Fluorescent Probe for the Detection of Carboxylesterase

Mengting Xia [1,2,†], Chunbin Li [2,†], Lingxiu Liu [2], Yumao He [2], Yongdong Li [1], Guoyu Jiang [2] and Jianguo Wang [2,*]

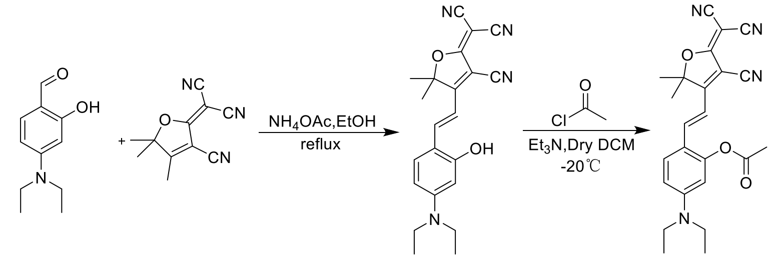

1 2 TCFIS TCFISE

Scheme S1. Synthetic route to TCFIS and TCFISE.

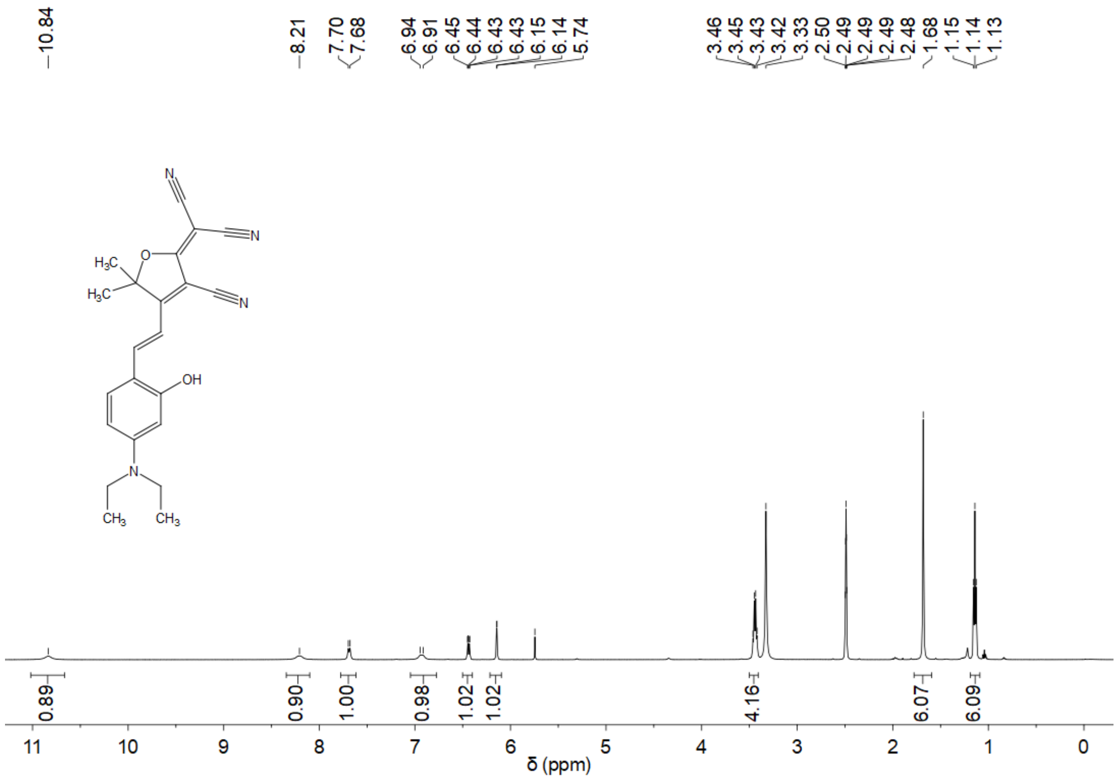

Figure S1. ^{1}H NMR spectrum of TCFIS in DMSO-d_6.

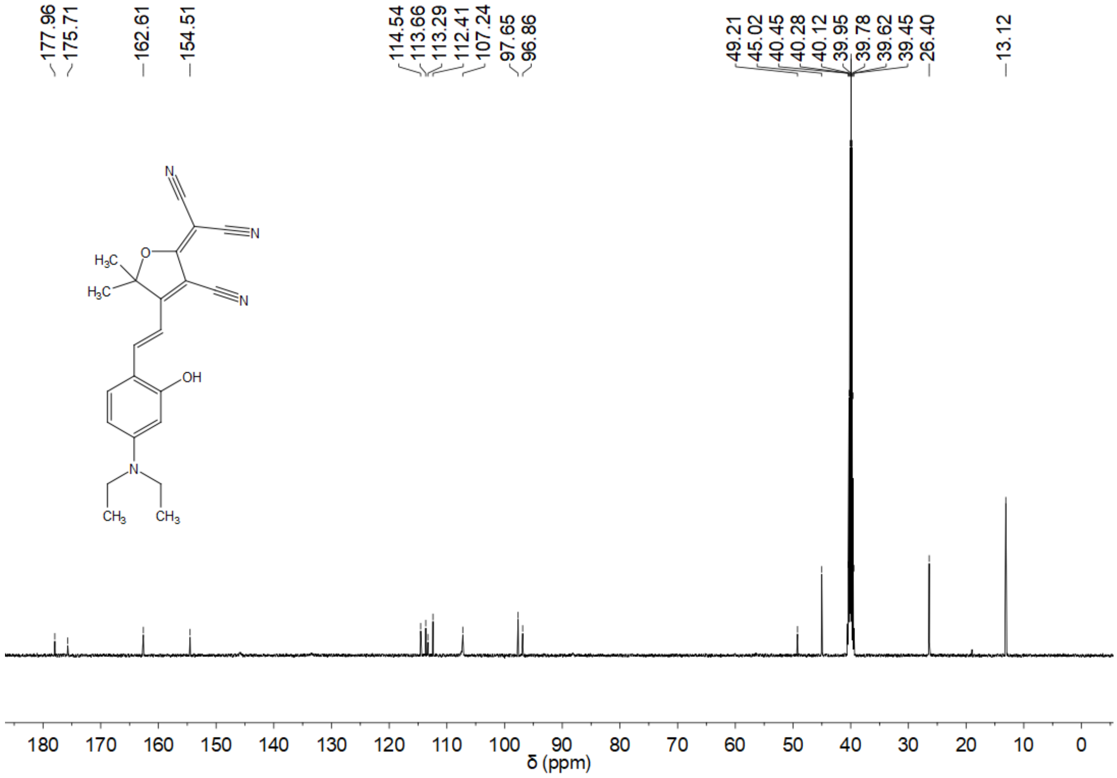

Figure S2. ^{13}C NMR spectrum of TCFIS in DMSO-d_6.

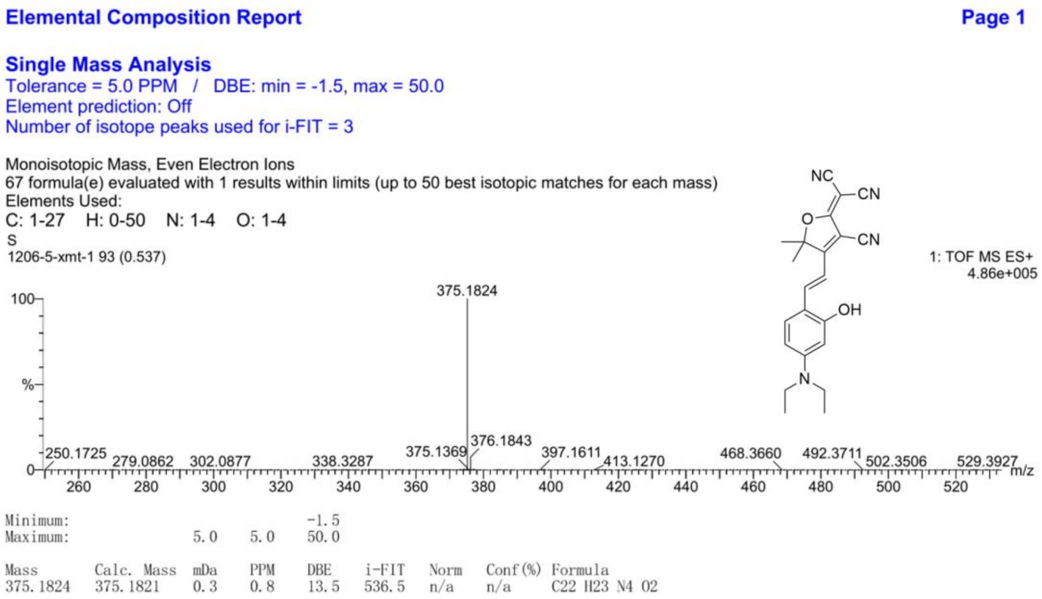

Figure S3. HRMS spectrum of TCFIS.

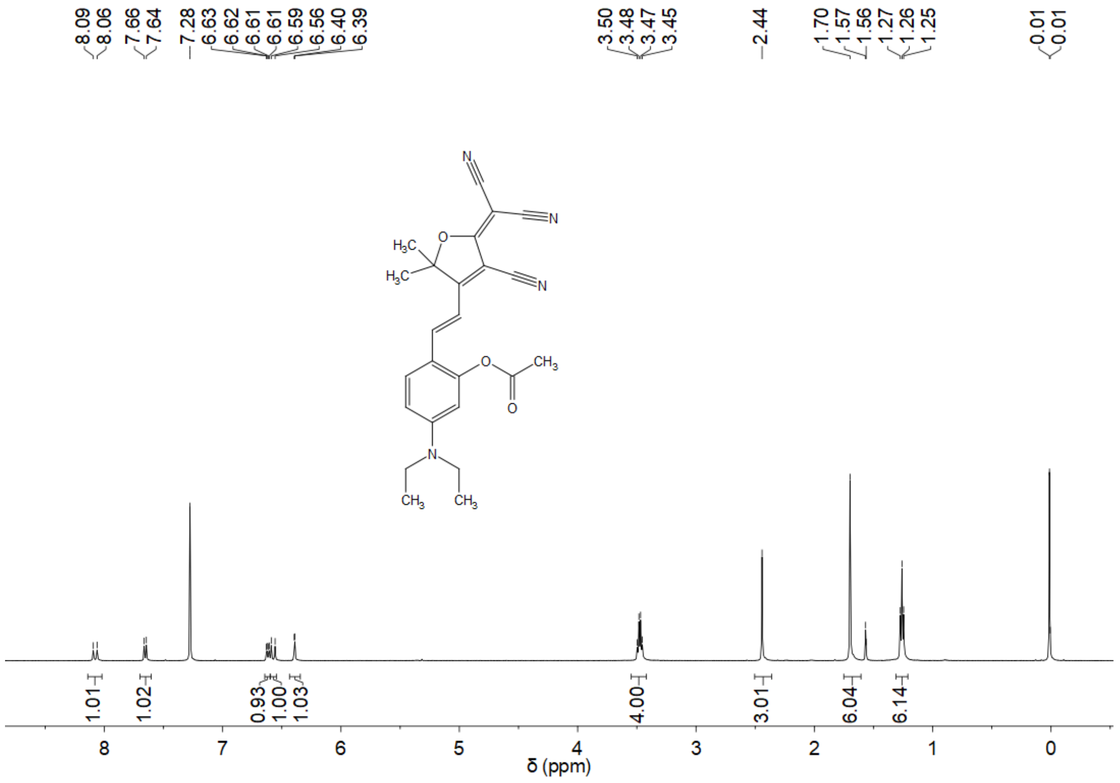

Figure S4. ^{1}H NMR spectrum of TCFISE in $CDCl_3$.

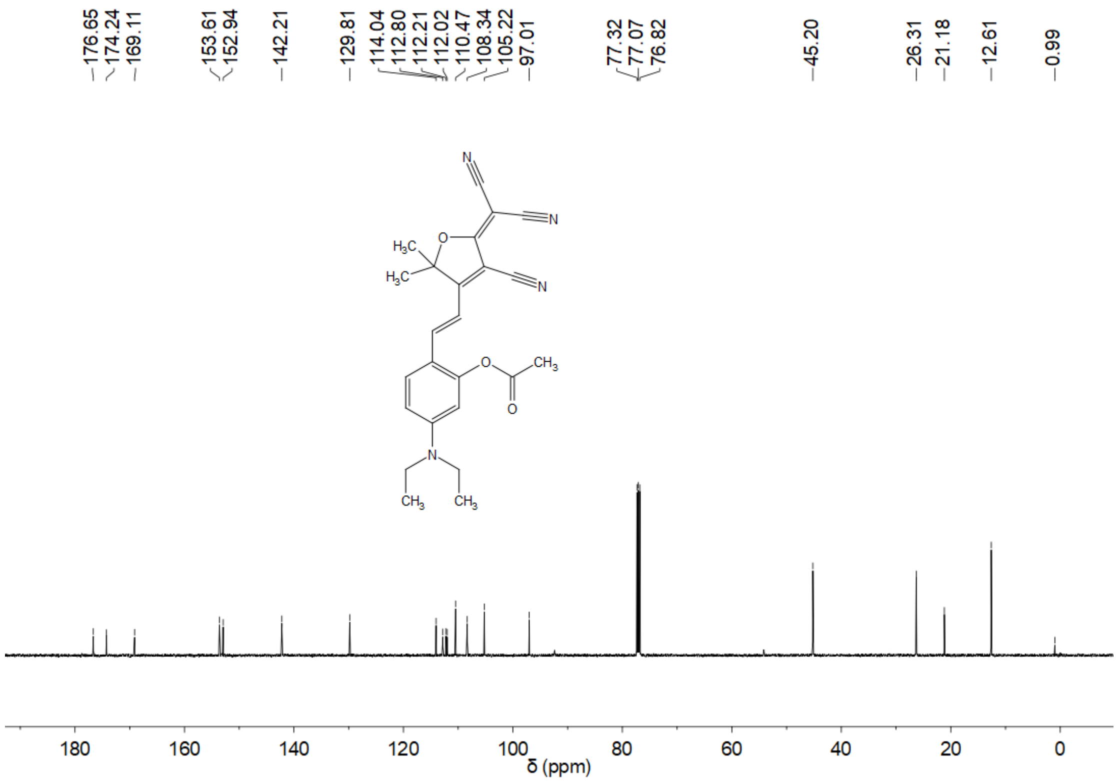

Figure S5. ^{13}C NMR spectrum of TCFISE in $CDCl_3$.

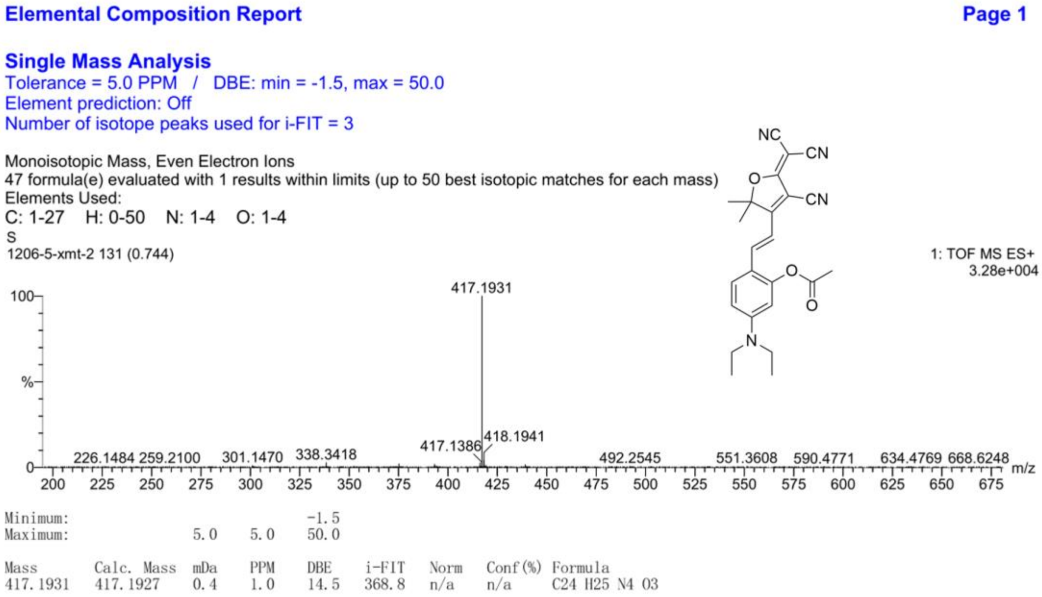
Elemental Composition Report **Page 1**

Single Mass Analysis
Tolerance = 5.0 PPM / DBE: min = -1.5, max = 50.0
Element prediction: Off
Number of isotope peaks used for i-FIT = 3

Monoisotopic Mass, Even Electron Ions
47 formula(e) evaluated with 1 results within limits (up to 50 best isotopic matches for each mass)
Elements Used:
C: 1-27 H: 0-50 N: 1-4 O: 1-4
S
1206-5-xmt-2 131 (0.744)

Minimum:				-1.5				
Maximum:		5.0	5.0	50.0				

Mass	Calc. Mass	mDa	PPM	DBE	i-FIT	Norm	Conf(%)	Formula
417.1931	417.1927	0.4	1.0	14.5	368.8	n/a	n/a	C24 H25 N4 O3

Figure S6. HRMS spectrum of TCFISE.

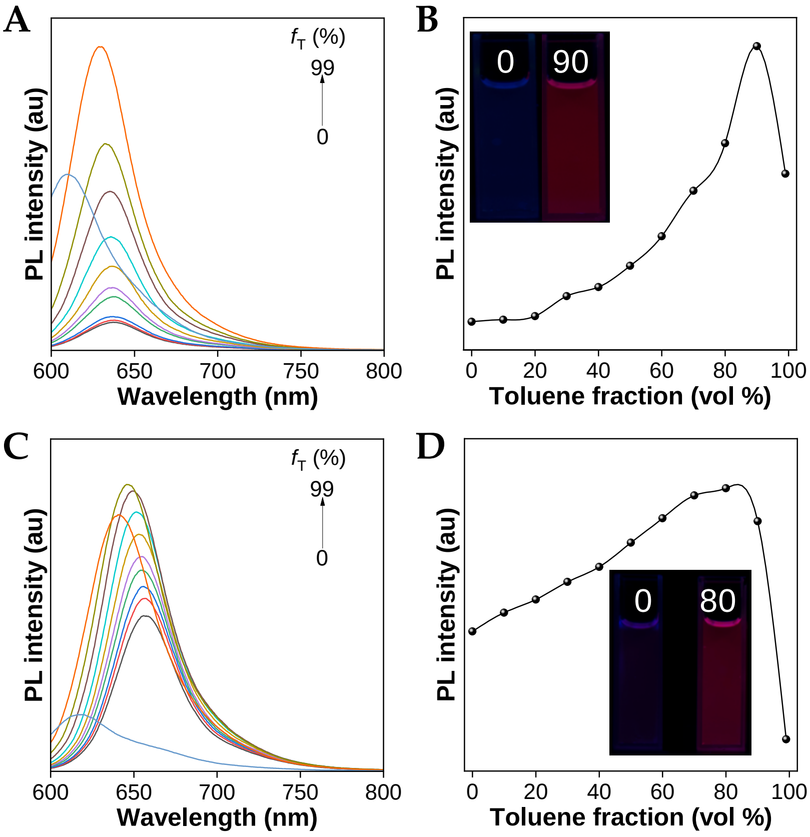

Figure S7. (**A**) PL spectra of TCFIS (5 μM) in toluene/DMSO mixed solution with different toluene fractions (f_T). (**B**) Plot of PL intensity of TCFIS (5 μM) at maximum emission wavelength versus the toluene fraction in the toluene/DMSO mixtures. (**C**) PL spectra of TCFISE (5 μM) in toluene/DMSO mixed solution with different toluene fractions (f_T). (**D**) Plot of PL intensity of TCFISE (5 μM) at maximum emission wavelength versus the toluene fraction in the toluene/DMSO mixtures. λ_{ex}=575 nm.

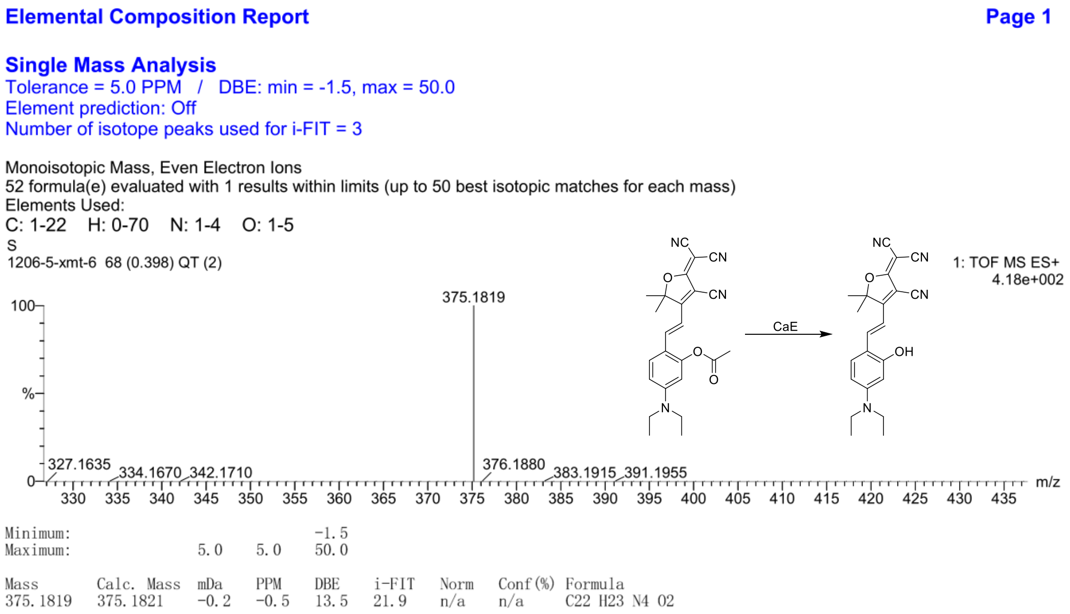

Figure S8. HRMS spectrum of TCFISE after incubation with CaE (1 U/mL) at 37 ℃ for 20 min.

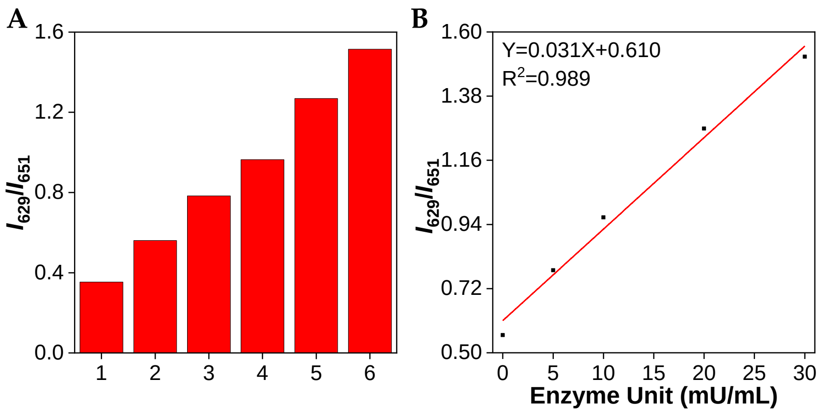

Figure S9. (**A**) Variation of the relative PL intensity ratios (I_{629}/I_{651}) of TCFISE in 20-fold diluted serum samples. 1: TCFISE only; 2-6: TCFISE + serum samples + CaE (0, 5, 10, 20, and 30 mU/mL). (**B**) Plot and linear fitting of relative PL intensity ratios (I_{629}/I_{651}) of TCFISE in 20-fold diluted serum samples vs the concentration of spiked CaE (0-30 mU/mL). λ_{ex}=575 nm.

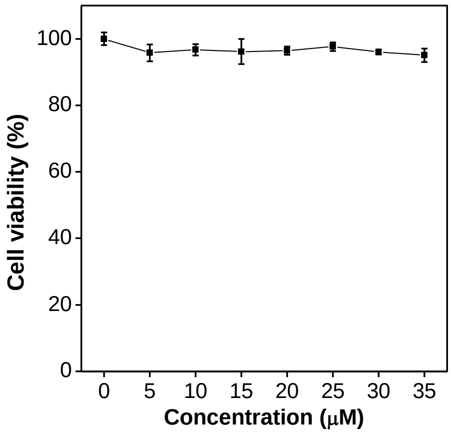

Figure S10. Cell viability of TCFISE toward HeLa cell by using MTT assays.

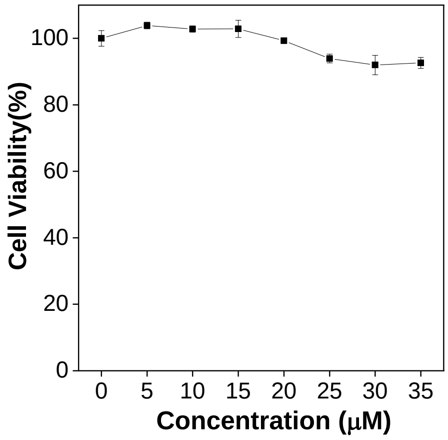

Figure S11. Cell viability of TCFISE toward HepG2 cell by using MTT assays.

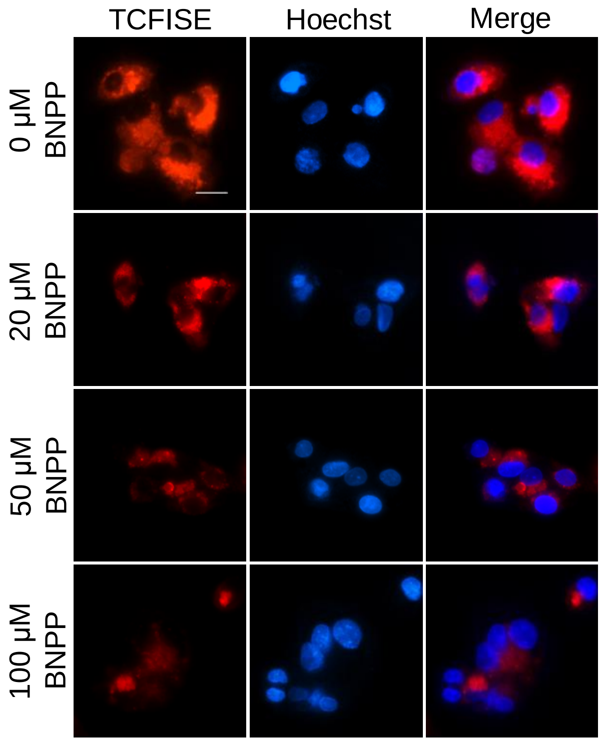

Figure S12. HepG2 cell imaging of TCFISE (5 μM) in the presence of different concentrations of BNPP. Commercial available Hoechst (1 μM) was used for the indication of nucleus. Scale bar: 20 μm.

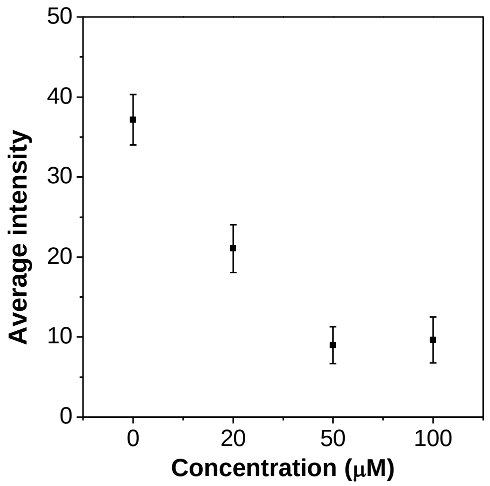

Figure S13. The average intensity of TCFISE channel in the presence of different concentrations of BNPP in Figure S12.
